# Nano Functional Food: Opportunities, Development, and Future Perspectives

**DOI:** 10.3390/ijms24010234

**Published:** 2022-12-23

**Authors:** Qijun Su, Xiang Zhao, Xin Zhang, Yan Wang, Zhanghua Zeng, Haixin Cui, Chunxin Wang

**Affiliations:** Institute of Environment and Sustainable Development in Agriculture, Chinese Academy of Agricultural Sciences, Beijing 100081, China

**Keywords:** functional food, nanotechnology, active ingredient, nano-delivery system, nano-nutrition additives

## Abstract

A functional food is a kind of food with special physiological effects that can improve health status or reduce illness. However, the active ingredients in functional foods are usually very low due to the instability and easy degradation of some nutrients. Therefore, improving the utilization rate of the effective ingredients in functional food has become the key problem. Nanomaterials have been widely used and studied in many fields due to their small size effect, high specific surface area, high target activity, and other characteristics. Therefore, it is a feasible method to process and modify functional food using nanotechnology. In this review, we summarize the nanoparticle delivery system and the food nanotechnology in the field of functional food. We also summarize and prospect the application, basic principle, and latest development of nano-functional food and put forward corresponding views.

## 1. Introduction

As the functional food industry is in the rapid development worldwide, the development and utilization of the functional food is getting more and more attention [1]. The use effect of functional food mainly depends on the effective utilization rate of the biological active ingredients. The stability, existing form, and usage of health functional foods have a great influence on the actual use effect of functional products. Although functional ingredients can be added to foods, they are very sensitive to external environments such as temperature and air, which exert a great effect on the smell, texture, and color of functional foods [2]. Active substances applied to functional foods mainly include phenolic substances, flavonoids (mainly flavonols, flavonoids, flavanols, flavanols, etc.), alkaloids, etc., and food pigment including β-Carotene, lycopene, lutein, and curcumin are also active substances [3]. All of them are natural antioxidants, which are easily affected by pH, temperature, light, and humidity. When active substances are added, the existing production process needs to be improved to maintain good physicochemical stability and prevent them from oxidative deterioration [4]. Therefore, appropriate release conditions can ensure the full use of such functional active substances in the human body [5]. Therefore, maintaining the biological activity of nutrient molecules and ensuring their full absorption in the human body will become the key in the field of functional food research.

Researchers are currently trying to find a way to improve food quality without compromising bioactive ingredients. The rapid development of nanotechnology and nanomaterials is gradually penetrating into various research fields around the world [6]. The materials with new properties and various functions are designed using nanotechnology at the level of atoms and molecules, which has played a major role in the field of medicine. In particular, a variety of intelligent and targeted nano-drug delivery systems are designed and constructed through nanotechnology and nanomaterials, which can be used to deliver drugs at the lesion location in a regular, quantitative, and fixed time to improve the efficacy and reduce side effects [7].

Nanotechnology and nanomaterials applied to the food field will be a new concept. The new properties and functions of nanomaterials can not only improve the food production process and production efficiency, but also produce many new functional foods with better functions and special functions, thereby improving the nutritional function, safety, and food quality [8]. The reduction of the size of nanoparticles can improve their bioavailability, including increasing the adhesion of active substances to tissues, improving the delivery efficiency of the gastrointestinal tract to active substances, reducing the impact of intestinal clearance mechanisms and prolonging the residence time of active substances in the gastrointestinal tract [9]. Nanoparticles not only affect the properties of functional food, but also determine the release rate of bioactive ingredients, reducing the side effects of the excessive intake of some functional food.

The development of nano functional food focuses on the two fields of nanoparticle delivery system and food nanotechnology. A nano-delivery system refers to the transportation of functional nutrients in the form of nano-micelles, nanoliposomes, nano-emulsion, nano-capsule, and nano-nutrient additive to play a role in the system. It is a kinetic, stable system with a targeted effect in vivo, which can increase the distribution of drugs in the lesion site, improve drug efficacy, and reduce side effects [10]. The ultra-fine nano powder extends the validity period of the active substance and improves its stability and safety through the controlled release of the carrier. Nano-nutrient additives can increase the direct absorption of trace elements for the human body into the intestinal tract and reduce the toxic and side effects in the body through the nano treatment. In this review, we summarize the existing technologies and achievements of the nanoparticle delivery system and comb the principles and methods of nanotechnology in improving the biological activity of functional food and promoting the release of its functional components, which can provide a reference for the application and development of nanotechnology in functional food.

## 2. Nanoparticle Delivery System

### 2.1. Nano-Micelles

**Figure 1:**
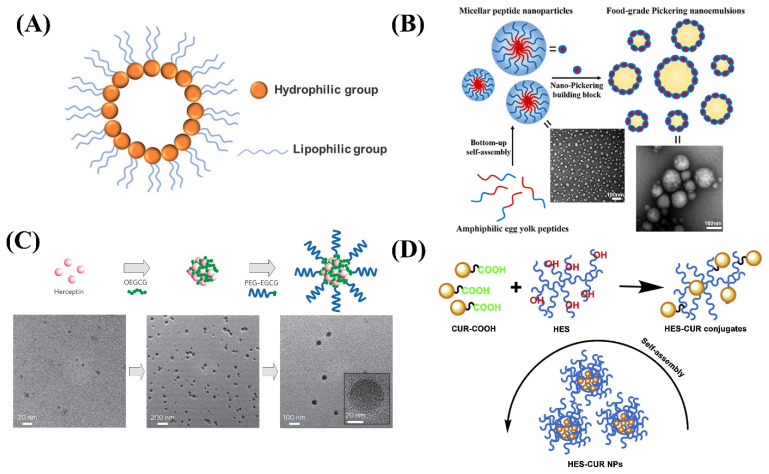
(**A**) The structure of nano micelles. (**B**) Schematic diagram of a spherical micellar nanoparticle self-assembled by yolk peptide. (**C**) Schematic diagram of the self-assembly process of the MNCs and TEM images of MNCs. (**D**) Schematic diagram for the formation process of the HES-CUR NPs.

A nano micelle belongs to the colloidal dispersion system; its particle size is smaller than liposomes and particles—generally a particle size of 5 to 100 nm (Figure 1A). In the low concentration of the aqueous phase, such amphiphilic molecules exist alone, but when the concentration reaches or is higher than its critical micellar concentration (CMC), the amphiphilic molecules in the aqueous phase form a stable micelle through self-assembly [11]. The drug carrier system of the polymer micellar hydrophobic core and hydrophilic shell has the advantages of insoluble drugs, improved bioavailability, targeted, and sustained release [12,13,14]. The most common micelle is the casein micelle in milk. Its biological function is to transmit minerals (calcium, magnesium, and phosphorus) from the mother to the larvae. In addition, it was used to carry amino acids and other nutrients for growth and development. Nano-casein micelles has been proved to play an important role as a carrier of hydrophobic bioactive compounds in research. These findings also support the use of casein micelles as encapsulation and delivery agents for hydrophobic bioactive agents in pharmaceutical and food industry applications [15].

Micellar systems are widely studied in the food production and biopharmaceutical field because of their ability to increase the bioavailability of drugs. Du et al. formed self-assembled nano-micelles from egg yolk peptides extracted from natural foods and applied them as emulsifiers (Figure 1B). The self-assembled nano-micelles had a smaller particle size, wettability, and an enhanced stability effect on Pickering’s milk, which remained sufficiently stable after spray drying and long-term storage [16]. Chung et al. prepared a self-assembling micellar nanocomposite consisting of green tea catechins and proteins (Figure 1C). Nano micelles formed by the self-assembling of EGCG derivatives and proteins in sequence can be well maintained intact and well stabilized [17]. Chen et al. coupled curcumin with food-derived hydroxyethyl starch to form nano-micelles (Figure 1D) and found that the solubility of curcumin nanoparticles was 1000 times higher than that of free curcumin molecules, and they can’t be damaged under light and high temperature conditions. The excellent properties of nano curcumin were proved by testing the drug loading rate, stability, and release rate [18]. Mohan et al. concluded that recombinant casein micelles in milk have the ability to act as nanocarriers or microcapsules, showing the ability of unmodified casein micelles to bind hydrophobic molecules such as vitamin A, curcumin, docosahexaenoic acid, and vitamin D. Experiments have been used to confirm that casein micelles in skimmed milk can be used as a carrier of vitamin A [19]. The selection of nano micelles systems in food is mainly influenced by its particle characteristics, loading characteristics, physicochemical properties, sensory properties, and economy. Nano micelles particles loaded with functional nutrients such as natural polyphenols, saponins, lipids, vitamins, etc. can be incorporated into functional food and beverage systems or converted into a powder form and used in supplements.

### 2.2. Nanoliposomes

**Figure 2:**
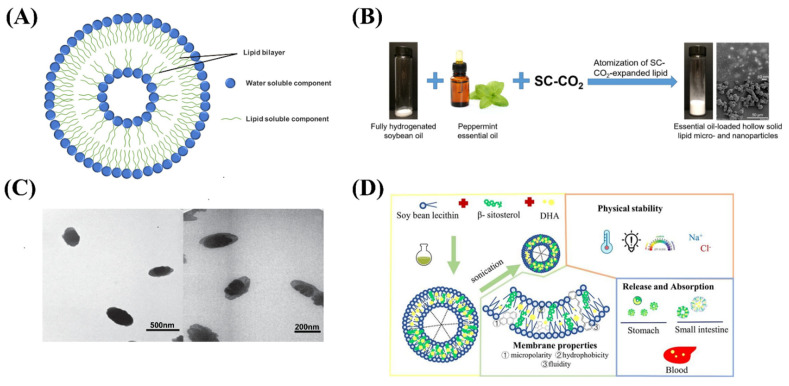
(**A**) The structure of nanoliposomes. (**B**) Schematic diagram of peppermint essential oil loaded hollow solid lipid micro- and nanoparticles. (**C**) TEM images of B12-loaded nanoliposomes. (**D**) Schematic illustration of DHA-loaded nanoliposomes.

Liposomes refer to a closed vesicle substance similar to a biofilm structure formed by phospholipids, cholesterol, etc. as membrane materials [20]. Solid lipid delivery systems are made by wrapping or interspersing active ingredients in lipid nuclei (Figure 2A and Table 1). Nanostructured lipid carrier (NLC) is a new type of lipid nanocarrier developed on the basis of liposomes, with particle sizes between 10–1000 nm. Nanoliposomes can improve the bioavailability of active ingredients in natural foods, reduce toxicity, and enhance gastrointestinal absorption [21,22]. Therefore, it is widely used in the research and development of improved and innovative health foods. In existing research, nanoliposomes are commonly used to encapsulate active ingredients such as lipids, antioxidants, proteins, vitamins, and minerals [23,24,25,26].

Essential oils are volatile and intensely irritating, and these problems limit their application in the food industry. Yang et al. develop free-flowing hollow solid lipid nanoparticles containing peppermint essential oils using carbon dioxide atomization (Figure 2B), which can achieve the highest essential oil loading efficiency at 50% initial essential oil concentration. Dry free-flowing products make essential oils easier to handle and store in the food industry, and simple and clean processes make scaling more feasible [27]. Baldim et al. prepared solid liposome nanoparticles of Indian lipid essential oil with a spray drying method. The essential oil solid liposome nanoparticles have higher thymol retention rate and rapid dispersion, which enhanced their stability to environmental factors, reduced their volatility, reduced toxicity, and increased bioavailability [28]. Raheleh et al. designed and optimized the microemulsion dilution methods of phenolic compounds encapsulated in liposomes, which can effectively maintain its stability and improve its bioavailability [29]. Tamjidi et al. prepared nanostructured lipid carriers with astaxanthin and applied them to beverage production, indicating that the functional beverages containing hydrophobic nutraceuticals has certain prospects and development value [30].

The nanoliposome embedding of vitamins and minerals has the potential to improve the bioavailability of fat-soluble substances and improve molecular release bias. Genç et al. produce solid liposome nanoparticles loaded with vitamin B12 with the final particle size of about 200 nm (Figure 2C), and its release rate was high [31]. Han et al. prepared DHA-loaded nanoliposomes by sonication method, which were stabilized by β-sitosterol (Figure 2D). The properties and membrane properties of DHA-NLs with different β-sitosterol contents were determined. The results showed that the maximum encapsulation efficiency of DHA-NLs encapsulated with soy lecithin and β-sitosterol was (86.95 ± 0.95) %. The particle size of all samples was within 200 nm and the relative retention was over 60% after 3 weeks of storage. Therefore nanoliposomes are expected to improve the absorption of DHA in ethyl ester form [32].

Some of the advantages of solid lipid nanoparticles over other lipid carriers are (1) the possibility of controlling drug release and drug targeting, (2) their stability under sterilization conditions, (3) their biodegradability, (4) protecting unstable compounds from chemical degradation, (5) binding hydrophilic and lipophilic drugs, and (6) the possibility of amplifying processes [33,34]. However, nanoliposomes composed of polar lipids (such as free fatty acids, surfactants, and phospholipids) are absorbed by epithelial cells as they have ester bonds that can undergo hydrolysis reactions. In addition, these liposomes can be hydrolyzed by the appropriate enzymes in the gastrointestinal tract [35], so we can’t predict whether this rapid hydrolysis process will cause toxicity when nanoliposomes are absorbed and accumulated in the gastrointestinal tract and in other organs. On the other hand, the oil phase of some nanoliposomes is not easily hydrolyzed by enzymes and is difficult to digest, which may lead to the absorption of the parts of the nanoliposomes rather than the active ingredient into the body, and its potential toxicity has not been elucidated.

### 2.3. Nano-Emulsion

**Figure 3:**
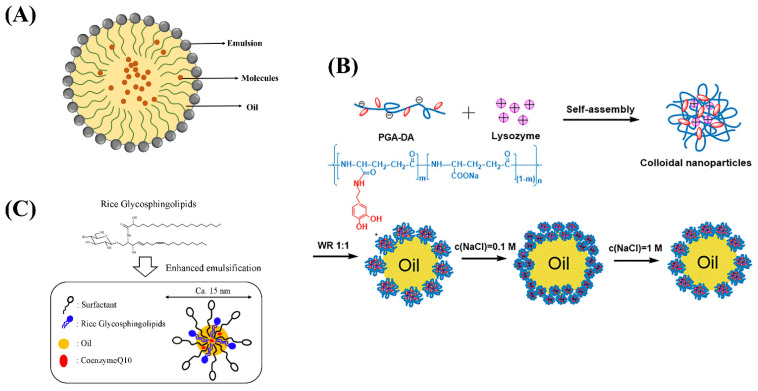
(**A**) The structure of nano-emulsion. (**B**) Schematic depiction of the formation and the oil-in-water interfacial behavior of Lys/PGA-DA colloidal nanoparticles. (**C**) Schematic representation of food grade microemulsion with rice glycosphingolipids.

Nano-emulsion is a thermodynamically stable oil/water mixing system, in which the effect of surfactant is transparent or translucent in appearance (Table 2 and Figure 3A) [36,37]. According to the dispersion between the two phases, microemulsions are generally divided into water-in-water (O/W) microemulsions, water-in-oil (W/O) microemulsions, and multiple emulsions (W/O/W) microemulsions [38,39]. Compared with traditional emulsions, nano-emulsions had many advantages that are beneficial to food processing [40]. Nanoparticles had a uniform size and good stability, avoiding aggregation caused by aggregation or gravity [41]. Second, the surfactant in the nano-emulsion was a biocompatible biological macromolecule, and the amount of surfactant was small. At the same time, the amount of organic cosolvent used in nano-emulsion was low, which improved the production efficiency [42,43,44,45].

In recent years, food products containing high levels of bioactive ingredients have become the mainstay of research and development. However, the poor water solubility, oil solubility, and stability of active substances limit their application in food system development. Nano-emulsions are used as carriers for high content bioactive ingredients to achieve their water or oil solubility and improve the dispersibility as well as bioavailability of the substances. Rao et al. prepared food grade microemulsions with lemon oil as the oil phase and sucrose monopalmitate as the emulsifier. The results show that microemulsions can be used as a carrier to evenly disperse functional factors in food [46]. Nano-emulsions are a new technology to modulate the main functional factors in fat-soluble foods, which improve its oxidation and instability [47]. Zhang et al. introduced lysozyme into dopamine-modified γ-glutamate and obtained self-assembled colloidal nanoparticles (Lys/PGA-DA) through electrostatic and hydrogen bonding interactions (Figure 3B). The colloidal nanoparticles were mixed with white oil to form o/w gel-like emulsions. It was shown that the ionic strength greatly improves the emulsifying property of colloidal nanoparticles. Colloidal nanoparticles are good emulsifiers for preparing gel like Pickering emulsions, which can improve the stability of microemulsion [48]. Khiew et al. prepared a water-in-oil (w/o) microemulsion system using biodegradable and non-toxic sucrose ester as a surfactant, and it can release the active substance through a temperature-controlled microemulsion system [49]. Kabir H. et al. studied the phase change during microemulsion formation and the effect of the main factor, temperature, on microemulsion changes, providing some basic theory for microemulsion research [50]. Natural plant-derived pigments have limited their use in the food industry due to their poor water solubility, susceptibility to oxidation, and poor stability. Jalali-Jivan et al. prepared lutein extracted nano-emulsion and optimized the extraction amount through ultrasonic pretreatment [51]. Roohinejad et al. prepared oil in water microemulsion and β-carotene extraction from pulsed electric field-treated carrot pomace was significantly improved [52]. Hou et al. constructed β-carotene emulsions and explored the effects of chitosan molecular weight on the stability and rheological properties of soybean soluble polysaccharide [53]. Qian et al. prepared nano-emulsion delivery systems and explored the effect of carrier oil on β- Effect of carotene bioavailability [54]. The above research opens up new avenues for the application of sensitive active ingredients in the food field.

In order to broaden the application of antioxidants in oils and fats, it is necessary to improve their solubility and stability in oils. Spernath et al. prepared lycopene microemulsions with ethoxylated sorbitan anhydride esters to improve their solubilization capacity and solubilization efficiency by adjusting the ratio of the aqueous and oil phases, which provided a new carrier system for the delivery of natural food supplements [55]. Yang et al. prepared aqueous oil-in-oil emulsion. When calcium and a phospholipid (DOPC) are added, the bioavailability of vitamin E can be improved [56]. Mao et al. prepared a multilayer emulsion of whey isolate protein–pectin and investigated the effects of pH, ionic strength, and artificial saliva on the stability of the emulsion [57]. Joung et al. prepared curcumin nano-emulsions with different concentrations of surfactants using a high-pressure homogenization method. The high concentration of surfactants improved the antioxidant capacity of Cur-Nes and retarded lipid oxidation. The experimental group can significantly reduce the degree of fat oxidation in milk compared with the control experiment, which confirmed the possibility that curcumin nano-emulsions can be used in dairy production [58]. Dasgupta et al. used edible mustard oil to prepare vitamin E acetate nano-emulsions with high antioxidant capacity and antifungal properties so that these nano-emulsions could be used in the production and processing of fruit juices to improve their shelf life, stability, and nutritional efficacy [59].

Although nano-emulsions offer an additional option for the delivery of active substances and can effectively increase bioavailability, safety and toxicity issues are still the first consideration. The small size effect of nano-emulsions allows them to pass rapidly through gastrointestinal barriers and cellular tissues so that additional substances for delivery of the active substance may accumulate in human tissues, and there is no way of predicting whether they will react with other substances and pose a potential safety risk. Toxicity studies of nano-emulsion delivered active ingredients are currently focused on in vitro simulations and animal studies and have not yet been carried out in clinical trials [60].

Solid nano-emulsions can encapsulate active functional factors in the oil phase as core materials and surfactants or other components as carriers. It can be made into powder, particle or tablet after drying and extrusion. The product was diluted with water to form transparent or translucent emulsion [61]. Conventional liquid microemulsions are not conducive to storage and transportation. In addition, the structure of microemulsion will also become unstable during long-term storage due to external factors such as temperature, pressure, and pH, thus affecting the encapsulation efficiency [62]. The solid microemulsions prepared by spray drying and lyophilization technology can be stored stably at room temperature for a long time. Uchiyama et al. investigated food grade microemulsion with rice glycosphingolipids (Figure 3C), which can enhance the oral absorption of coenzyme Q10 and provide a new idea for the absorption of functional active substance delivery [63].

**Table 2:** Examples of nano-emulsions for functional food nanoparticle delivery systems.

Materials	Method	Active Components	Size (nm)	Zeta-Potential (mV)	Application	References
Sodium dodecyl sulfate	Ultrasonic emulsification	Lutein	89.6	−16.6	Food colorant	[51]
Glycerol monocarpy locaprate	Emulsification	β- carotene	74.02	−37	Food additives	[52]
Chitosan	High pressure homogenization method	β- carotene	520	55	Food additives	[53]
MCT, LCT	High pressure homogenization method	β- carotene	150	−36	Gastrointestinal tract delivery carrier	[54]
Sorbitol	Emulsification	Lycopene	-	-	Antioxidant	[55]
LCT, MCT	homogenization method	Vitamin E	145	−63.7	Antioxidant	[56]
Sunflower seed oil	homogenization method	Pectin	204.3		Stabilizer	[57]
MCT, LCT	High pressure homogenization method	Curcumin	90		Antioxidant	[58]
Mustard oil	Phase separation technique	Vitamin E	86.45		Antioxidant	[59]
Rice Glycosphingolipids	Freeze drying	CoenzymeQ10	20		Food additives	[63]

### 2.4. Nano-Capsule

**Figure 4:**
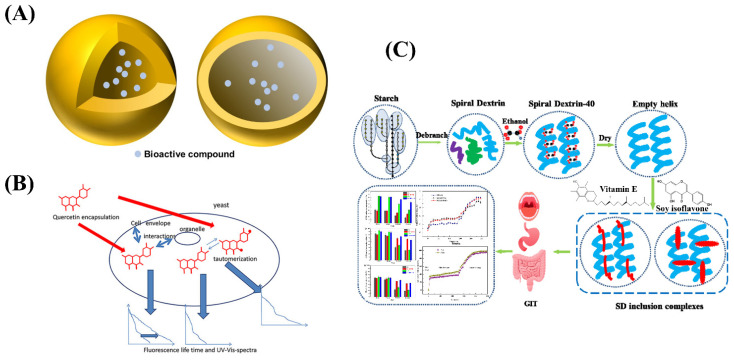
(**A**) The structure of nano-capsule. (**B**) Schematic illustration of quercetin encapsulated in yeast microcapsules. (**C**) Schematic diagram of nanocapsule of vitamin E and soy isoflavone using spiral dextrin.

In recent years, nano-capsule technology has become a hot spot in the field of food research and application. Nano-capsules are nano sized polymer microcapsules (Table 3 and Figure 4A), which are formed by encapsulating solid, liquid, or even gas active substances with natural or synthetic polymer materials. Nano-capsules are small in size, easy to disperse, and suspend in water and form a homogeneous and stable colloidal solution. In addition, it has good targeting and sustained release effects [64,65]. In the field of functional food, nano-capsule technology was used to embed functional factors in functional foods, which can not only reduce the loss of functional factors during processing or storage, but also effectively deliver functional factors to the gastrointestinal tract of the human body [66,67,68,69]. The specific targeting of nano-capsules can change the distribution of functional factors and concentrate them in specific target tissues to achieve the purpose of reducing toxicity and improving efficacy, and they can improve their bioavailability by controlling the release of functional factors while maintaining the texture, structure, and sensory attractiveness of the food [70,71,72,73,74].

The wall materials of nano-capsules should meet the requirements of non-toxic, edible, good biocompatibility, and degradability in the food industry, and at the same time, the properties of core materials and wall materials and the application performance of microcapsules should be comprehensively considered [75]. In functional food, the core material is mainly the functional factor with biological activity, and the wall material is mainly the environment-friendly composite material with biodegradability. There are some problems in the practical application of traditional wall materials, such as that natural polymer materials have poor mechanical strength and unstable quality, semi-synthetic polymers with poor acid resistance and high temperature resistance are prone to hydrolyze, and synthetic polymer materials have certain toxicity and high cost [76]. The wall materials used in food are mainly composed of carbohydrates, plant water-soluble gums, and proteins. Because they have their own advantages and disadvantages in practical application, three types of wall materials are often used in the form of mixture to improve their performance [77].

In order to address the application of fat-soluble active substances in the food field and enhance their stability, antioxidant properties, and use effect, Prakash B et al. addressed the challenges of plant essential oils in the food industry and explored the potential application of plant essential oil nano-capsules as a new generation of food preservatives [78]. Prasad et al. encapsulated coffee bean oil in nano-capsule wall materials such as β-cyclodextrin or whey protein, which exhibited thermal stability and antioxidant properties [79].

Nanoencapsulation technology is usually used to embed the sensitive active ingredients in functional food so as to protect them from the impact of external environment conditions (including pH, light, temperature, etc.) and keep them highly bioactive. Liu et al. prepared novel lactic acid bacteria nano-capsules with sodium alginate as the encapsulating material and explored the physical properties of the nano-capsules and their protection against lactic acid bacteria in a simulated gastrointestinal environment, which provided a new idea for developing a feasible and stable preparation method of microbial particles [80]. Chen et al. constructed the anthocyanin microcapsules drug delivery system and characterized the physicochemical properties, pharmacodynamic properties in vivo, targeting mechanism, cell internalization, and release kinetics of nano-capsules [81]. Sharif et al. investigated the preparation process of anthocyanin nano-capsules, explored the effects of different carrier components and encapsulation techniques on the preparation of nano-capsules, and finally found that carbohydrates such as maltodextrin and gum are the most commonly used polymers for encapsulating anthocyanins [82]. These studies open up new avenues for the application of sensitive active ingredients in the functional food field.

At the same time, researchers have developed new wall materials such as liposomal wall materials [83], microbial wall materials [84], and modified wall materials (such as modified starch, modified chitosan) [85,86]. Liposomes have good biocompatibility and targeting and can also embed water-soluble or water-insoluble core materials [87]. Dai C et al. prepared a new type of bovine serum albumin nano-capsules using sodium alginate liposomes and confirmed its good protein delivery properties. The effects of liposome composition and multivalent cations on the morphology, encapsulation efficiency, integrity, and in vitro release characteristics of the nano-capsules were explored [88]. Microbial microcapsules were used to encapsulate active components with fungal microorganisms such as molds and yeasts as carrier materials so that substances can freely penetrate cell walls and cell membranes into cells [89]. In yeast microcapsules, yeast cells, as a good wall material for preparing microcapsules, have the characteristics of rich sources, safety, non-toxicity, biodegradability, uniform particle size, and the ability to ensure its safety in food applications. Pham-Hoang et al. constructed quercetin microcapsules with yeast cells (Figure 4B), which exhibited excellent stability relative to drug single molecules [90]. Zhang Z et al. prepared thyme essential oil nano-capsules using chitosan and sodium alginate as wall materials with a layer-by-layer encapsulation method and explored the antibacterial properties of thyme oil nano-capsules under different temperature and pH conditions. The optimal conditions were obtained by comparing the size of the inhibition circle size and inhibition rate. It was also verified that the thyme essential oil has a good inhibitory effect on Staphylococcus aureus, which has the potential to become a good antibacterial agent in the food and pharmaceutical industries [91].

**Table 3:** Examples of nano-capsules for functional food nanoparticle delivery systems.

Materials	Method	Active Components	Size (nm)	Advantages	Application	References
Whey isolated protein	Spray drying	Roasted coffee soybean oil	315.4	Prevents oxidative rancidity of coffee soybean oil.	Food flavoring	[79]
Sodium alginate, chitosan	Ultrasonic mothed	Orange essential oil	-	Encapsulation rate increased from 53.8% to 74.4%.	Food additives	[80]
Sodium alginate	Reversed-phase evaporation technology	Bovine serum albumin (BSA)	-	Protects BSA from destruction, improves bioavailability.	Improves protein stability	[88]
Sodium alginate, chitosan	Self-assembly method	Thymol essential oil	71.1	Good pH responsiveness, The antimicrobial ring was reduced from 18.5 ± 0.6 mm to 12.3 ± 0.6 mm	Food antimicrobial	[91]
Octenylsuccinic anhydride (OSA)	Spray drying	Vitamin E	235	Enhances VE stability and solubility, protecting it from destruction	Food additives	[92]

The modified wall material can improve its poor water solubility and has good biocompatibility and film-forming property, so it has a good application prospect. Hategekimana et al. prepared vitamin E nanocapsules by spray drying using octenyl succinic anhydride (OSA)-modified starch as a wall material. The effects of physicochemical properties of modified starch on the emulsifying ability and storage stability of vitamin E in nano-capsules were studied [92]. Fu et al. co-encapsulated vitamin E (VE) with coenzyme Q10 using whey protein isolate (WPI), WPI/soluble corn fibre (SCF), and WPI/maltodextrin as wall materials, which exhibited a high retention rate of active substances and antioxidant activity [93]. Wang et al. prepared V-shaped inclusion complexes through the interaction of modified dextrin and vitamin E (Figure 4C), which showed a controlled release and sustained release behavior during digestion. In addition, the inclusion complex has higher stability and stronger antioxidant capacity than a single component [94]. The above results lay a foundation for the study of a drug loading system of functional active substances.

### 2.5. Nano-Nutrient Additive

Nano trace element additives are new active ingredients obtained by the nano processing of essential trace elements for human body, which can improve the physical and chemical properties, solubility, oxidation resistance, retention release time, and stability in the gastrointestinal tract. This method can strengthen the use of trace elements in food and the absorption effect in the body and can improve their bioavailability and safety (Table 4).

#### 2.5.1. Nano Selenium

Selenium is an essential trace element for the human body. Nano selenium has better absorption and less toxicity compared to traditional selenium supplements. The research on nano selenium additives is of great significance for selenium deficient people and clinical use.

Nano selenium is usually encapsulated in polysaccharide carrier materials such as chitosan, glucomannan, arabic gum or carboxymethyl cellulose. Zhang et al. designed a method to encapsulate selenium into chitosan nanoparticles. This study showed that the encapsulation of selenium compounds into chitosan nanoparticles was an effective way to deliver selenium to cells, which was conducive to increasing selenium retention, improving the immune system, and reducing the risk of DNA damage [95]. Cheng et al. found that a proper selenium supplementation can improve the levels of tumor necrosis factor-α (TNF-α) and interferon-γ (IFN-γ) in the body, thereby improving the immune response to the influenza ANWS/33 (H1N1) virus [96].

#### 2.5.2. Nano Iron

**Figure 5:**
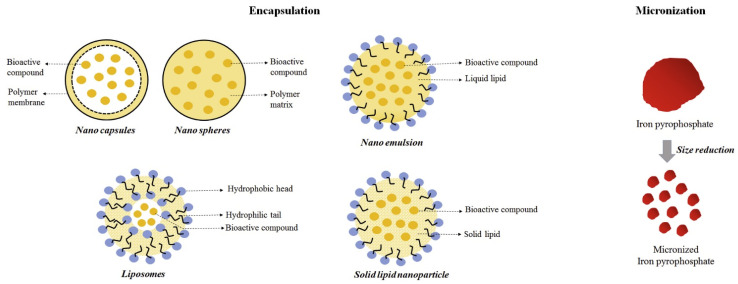
Schematic diagram of encapsulation and micronization approaches for iron fortification.

Iron salt fortified food can provide the safest, most effective, and most convenient iron supplement method for iron deficient people (Figure 5). At present, most iron complexes (ferrous sulfate and ferrous chloride) are highly water-soluble, but they can cause unacceptable sensory color changes and gastrointestinal problems. Insoluble iron salt (ferric pyrophosphate) in food excipients does not cause unacceptable taste or color, but its bioavailability is low. Nano iron can effectively avoid the above problems and solve the global iron deficiency anemia problem.

Shubham et al. stated that nanometer sized ferrous particles were released and oxidized into iron on plasma membrane of enterocytes through iron transporters in vivo. The nano iron was transported to the active site by binding with hephaestin proteins [97]. Hurrell et al. reported the particle size of iron pyrophosphate was reduced from 8 μm to 4 μm, and the absorption rate of iron by adults was increased by 2–4 times [98]. These studies showed that reducing the particle size was an effective strategy for improving iron bioavailability. The particle size reduction technique increases the surface area of iron compounds, thereby improving their solubility in gastric acid, leading to higher absorption. Salahuddin synthesized ascorbic acid-coated spherical iron oxide (Fe_3_O_4_) nanoparticles in the size range of 20 ± 2 nm. Fe_3_O_4_ fortified biscuits with different concentrations were made. The experimental results showed that the experimental group taking iron fortified biscuits could recover completely from anemia within five weeks without any toxic symptoms and mortality [99]. In addition, Yun et al. [100] orally administered nano iron oxide particles to rats every day for 13 weeks. The toxicity study results showed that there were no residual particles in the liver, kidney, and other tissues in the body, which would not produce aggregation and toxicity.

#### 2.5.3. Nano Zinc

Nanometer zinc is an essential trace element for the human body and an essential element to form a variety of human proteins. Nanometer zinc has high activity, a high absorption rate, immunity regulation, and other functions. Alkhtib et al. found that there is potential to use nano form to transport zinc to improve bioavailability and digestibility. The experiment showed that amino acid coated zinc improved the growth and feed consumption of poultry and also improved the digestibility of zinc [101]. Dukare et al. found that 80 ppm dietary nano zinc can significantly reduce the content of cholesterol and fat in meat, improve the free radical scavenging capacity, reduce the lipid peroxidation of meat, improve the activity of serum antioxidant enzymes, promote bone growth and development, and increase the accumulation of zinc in the body [102]. It is important to note that zinc oxide nanoparticles can penetrate into cells and produce reactive oxygen species, which can cause damage to the cell structure and thus produce cytotoxicity. Therefore, the dosage of zinc nanoparticles as nutritional additives in food should be strictly controlled to prevent excessive intake and absorption into the human body, which can avoid health risks [103].

#### 2.5.4. Nano Calcium

Calcium has a wide range of important physiological and biochemical functions in organisms; it can promote bone development, maintain the normal structure of cell membrane, and maintain the normal function of the nervous system. However, as ordinary calcium is insoluble in water, it is not easy to be absorbed and utilized by the human body when eaten. Insufficient food intake will lead to insufficient calcium level in the human body, which will affect human health. Nanometer calcium carbonate has a low production price and high calcium content. It can significantly improve the calcium nutritional status of the human body when added to food. Therefore, nanometer calcium carbonate is a good source of calcium supplements, which can be used as food additives and calcium fortifiers [104].

**Table 4:** Mechanism and application of nano-nutrient additives.

Materials	Mechanism	Applications	References
Nano Selenium	Dependent on the synthesis and expression of selenoprotein.	Food additives, Feed additives	[96]
Nano Iron	Loading and transporting iron ions to various tissues of the body.	Nutritional enhancers, food additives	[99]
Nano Zinc	Absorption of zinc transporters on cells through the intestinal mucosa.	Food packaging materials, feed additives	[102]
Nano Calcium	Calcium ions enter the blood circulation through the epithelial cells of the small intestine.	Nutritional enhancers, food packaging materials	-

## 3. Functional Food Nanotechnology

Functional food nanotechnology refers to the food processing technology that uses nanotechnology and nanomaterials to modify, package, and deliver active ingredients in the production process. The technology greatly improves the bioavailability, targeting, and controlled-release in the intestine [105]. At the same time, the application of nanotechnology in functional food can also improve the appearance and taste of food and enhance the suitability of the product; therefore, the food nanotechnology has great research importance [106]. Nanotechnology is widely used in the food industry and can be divided into bottom-up and top-down processing according to the preparation method (Table 5).

### 3.1. Bottom-Up Processing Technology

Bottom-up nano processing methods include self-assembly, nanoprecipitation, phase separation techniques, and template method. Biodegradable materials, such as chitosan, protein, and pectin, can modify and encapsulate nutrients to form food nanomaterials.

**Figure 6:**
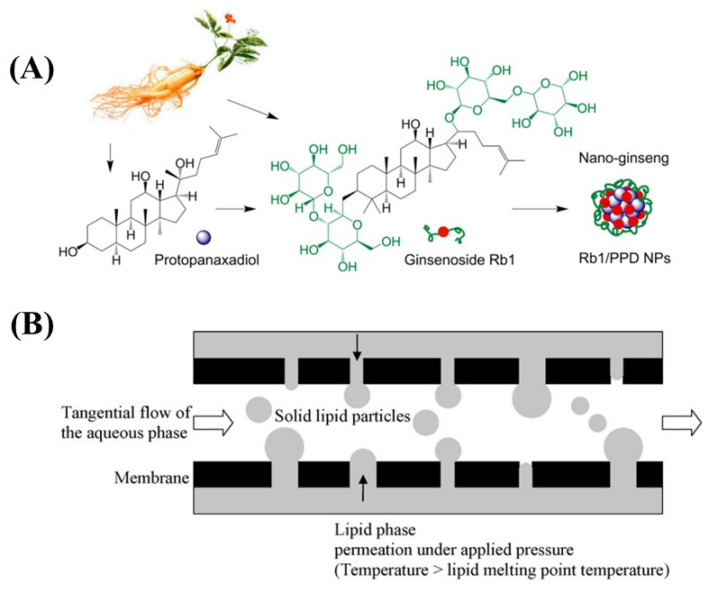
(**A**) Illustration of ginsenoside nanoparticles formed by self-assembly method. (**B**) Schematic drawing of the solid lipid particles by membrane emulsification.

The principle of molecular self-assembly is to form a class of molecular aggregates or supramolecular structures through covalent interactions using molecular recognition between molecules. Molecular self-assembly has clear and stable structure and specific properties [107]. Zhou J et al. prepared spherical nanoparticles encapsulating astragaloside with a diameter of 74.1 ± 0.7 nm and a zeta potential of −24.3 ± 1.7 mV using a self-assembly method, which showed low cytotoxicity and hepatocyte gain effects in cellular experiments [108]. Dai et al. used a self-assembly method to design and prepare a novel “ginseng nanoparticles” with well-defined composition (Figure 6A). The optimized nanoparticles exhibited smaller size, higher drug loading and encapsulation rates, and better in vitro and in vivo antitumor effects. The process of self-assembled nanoparticles has high biocompatibility, and the preparation process is simple, mild, and free of organic solvents and cross-linking agents [109].

The main method used in food is aqueous phase separation. The principle is to evenly disperse the core material in the wall material solution and then add another substance or solvent to condense the wall material mixture and form microcapsules around the core material [110]. Li et al. successfully used sodium alginate and chitosan to microencapsulate yolk immunoglobulin and found that sodium alginate/chitosan microcapsules can effectively protect yolk immunoglobulin from the killing effect of gastric juice [111]. The phase separation method is a very effective microencapsulation technology with a relatively simple process, and the embedding rate is 85%~90%.

Template synthesis is a preparation process that uses a material with nano structure as a template, and the active substances are physically or chemically deposited into the pores or surface of the template, and then the template is removed for synthesis. The shape of the nanomaterial is easy to control and obtain [112]. Zhang B et al. combined ursolic acid and gallocatechin gallate (EGCG) from green tea into nanoparticles. First, ursolic acid was simply self-assembled to form nanoparticles, and then EGCG was coated on the surface of nanoparticles through intermolecular hydrogen bonds and hydrophobic interactions to form core-shell structure nanoparticles. Nanoparticles enhance the preservation and release of active ingredients, activate the immunity of the body, and produce effective anti-tumor effects in vitro and in vivo [113].

Several common bottom-up methods can be effectively modified and embedded in functional food processing, and some unstable and easily oxidized ingredients can be processed into more stable nanoparticles, which can achieve a greater release of active substances, improve bioavailability, avoid waste, and reduce economic costs.

### 3.2. Top-Down Processing Technology

Top-down nano processing methods include grinding, high pressure homogenization, ultrasonic emulsification, membrane emulsification, and supercritical fluid. The top-down processing technology is to use nano crushing technology to process active components into nano sized substances, so that the components show unique physical and chemical properties when added to food.

Grinding is a traditional method that can mechanically crush active ingredients into nanoscale particles with physical action. For example, the grinding technology can be used to prepare fine grain wheat flour with a low moisture content [114] and can also be used to enhance antioxidant activity in green tea powder [115]. Homogenizers are often used to reduce the size of fat balls to increase the stability of the emulsion [116]. Ramachandraiah et al. investigated the effect of ground celery powders (CPs) on the physicochemical, antioxidant, and antimicrobial properties of meat products. Compared with the pork without CPs, the addition of ultrafine CPs powder in pork has a tendency to increase the antioxidant activity [117]. Wu et al. crushed the Panax notoginseng flowers with the ultra-micro grinding technology and found that the mechanical shear stress played a crucial role in the decomposition process of Panax notoginseng flowers. The size of particles decreased significantly, and the content of active ingredients such as saponins, minerals, total phenols, and flavonoids in Panax notoginseng flowers increased [118].

High pressure homogenization uses high mechanical energy to a provide strong splitting force to act on liquid and finally form very fine lotion droplets. In general, high-pressure homogenizers are better at producing finer lotion than traditional homogenizers [119]. In addition, high-pressure homogenization in functional food can improve the safety and shelf life of food products. Xu et al. used high-pressure homogenization to prepare nano-emulsions of soy protein and the output products performed well in terms of heat treatment and storage [120]. Akbas et al. used high-pressure homogenization to prepare capsaicin nano-emulsions. The capsaicin nano-emulsions with good antibacterial activity and physical properties such as particle size and color were obtained [121].

Ultrasonic emulsification is a form of homogenization for liquid foods, which can reduce particle size and enhance texture and taste [122]. Falsafi et al. produced oat starch nanoparticles with a size of 48–63 nm under ultrasonic condition of 85% amplitude, 500 W power, and 25 °C [123]. The albumin nanoparticles with a particle size of less than 200 nm have been used effectively for the production of salad dressings, creams, yoghurts, syrups, chocolate and malt beverages, oil emulsions, fillings, and frostings [124]. Ultrasonic emulsification is also used to prepare oil and water nano-emulsions using high intensity ultrasound waves, which can modify the characteristics of the treated substances through intense shear, pressure, and temperature [125].

Micro-fluidization means that the fluid is forced to pass through the microchannel under high pressure (500 to 20,000 psi). The micro-fluidization allows the mixing of water phase and oil phase at the micro level and then passing them through the microfluidic device. High speed collision of emulsion generates shear force, cavitation force, and impact force, and then it generates stable nano-emulsion [126]. Zabihi et al. prepared curcumin nanoparticles encapsulated in poly (lactic acid) using micro-fluidization and ultrasonication to significantly increase the drug loading of the nanoparticles. The prepared curcumin nanoparticles exhibited sustained release properties [127].

There are two main types of membrane emulsification including direct membrane emulsification (DME) and premixed membrane emulsification (PME). DME is to introduce dispersed phase into stirred or cross flowing continuous phase through microporous membrane to produce nano-droplets [128]. PME is introduced into the prepared coarse lotion (premix) through the porous membrane to form droplets (Figure 6B). D’oria et al. used the membrane emulsification equipment to press the lipid through the membrane hole to produce small droplets under high temperature. These droplets are taken away by circulating water to form stable and uniform solid lipid particles [129].

Membrane emulsification has lower energy input compared with other emulsification methods, which leads to a lower working temperature during the emulsification process and improves the stability of sensitive substances. Membrane emulsification is a new technique for the preparation of emulsions with a small particle size and narrow distribution and is seldom used at present.

Supercritical fluids are fluids with temperatures and pressures higher than their critical temperatures and pressures, which have the characteristics of both gases and liquids. The most commonly used preparation technique is the rapid expansion of a supercritical solution. The solute dissolved in supercritical carbon dioxide and was rapidly sprayed into a low-pressure container through a nozzle to form nanoparticles. At the same time, a large number of high-pressure CO_2_ molecules collided with the pronucleus in the process of particle formation, which can prevent the aggregation and growth of particles [130]. Shin et al. prepared vitamin K nanoparticles by supercritical fluids technology and investigated the influence of the preparation conditions on the experimental results. They concluded that the geometry of the nozzle, the injection rate of the solution, the pressure of CO_2_, and the type of organic solvent determined the particle size and crystal shape of the nanoparticles prepared by supercritical fluids technology [131].

**Table 5:** Advantages and disadvantages of common food processing nanotechnologies.

Methods	Advantages	Disadvantages	References
Grinding	It has lowest cost, the most widely used technology, its simple operation and suitable for continuous production.	It has certain requirements for wall materials and is not suitable for nano-thermal materials.	[114]
High pressure homogenization	Good emulsification performance and efficiency.	Consumes a lot of vegetable oil and has a high cost of use.	[119,120]
Phacoemulsification	Improved bioavailability, suitable for heat-sensitive substances.	The power consumption is large and takes a long time.	[122]
Microfluidics	Narrower particle size distribution vs. smaller particle size.	Special devices are required, and the process is more complicated.	[126]
Membrane emulsification	Low energy consumption has good industrial prospects, and can achieve a closed and sterile environment for pipelines.	The membrane flux needs to be improved, and the membrane cost is high.	[128]
Supercritical fluids	No solvent residue, low cost, environmentally friendly.	The corresponding solubility of the material is required.	[130]

The bottom-up methods generally reduce the particle size of active substances through external forces, resulting in different functions due to changes in their specific surface area, appearance, and shape. The bottom-up method is widely used in food processing because of its simple operation, short cycle, and low labor cost.

## 4. Summary and Outlook

Functional food plays an important role in ensuring the balanced nutritional needs of the human body and maintaining health compared with ordinary food. The improvement of the effective utilization of bioactive ingredients in functional food is the key to the development of functional food. Based on the development of nanotechnology, researchers have studied the nano-delivery system of active substances and the nano processing technology of functional food. A nanoparticle delivery system can target or control the release of active ingredients using nanotechnology and nanomaterials. Small size effect, pH response characteristics, and charge change were used to promote the absorption and adhesion of active substances, ensure the activity of substances, and improve the bioavailability. Functional food nano-components can reduce their particle size, increase the adhesion of active substances to tissues, improve the delivery efficiency of active substances, and extend the retention time of active substances in the gastrointestinal tract and improve their bioavailability by reducing the impact of an intestinal clearance mechanism and increasing the surface activity of active substances. Through various characterization experiments reported in the literature, it is proved that these nanoparticle delivery systems can improve the biological activity stability of active ingredients, and their release mode is scientific, which provides a basis for their development and application in functional food.

This paper first analyzes the challenges and difficulties in the delivery process of nano-functional foods and summarizes the optimal solution to such problems—the nanoparticle delivery system. We introduce several common delivery method;, list recent related research; summarize the analogy of materials, methods, and experimental results; and analyze their effectiveness, safety, and development potential. Then, the common technologies in the above delivery systems are summarized, and their advantages, disadvantages, and actual performance are analyzed and compared. Although functional food nano delivery technology has made some progress, the shortcomings of current nanoparticle delivery systems are also worthy of attention. The safety of materials used to construct nanoparticle delivery systems needs to be further verified while their carrying capacity and application score are still very small. Although a large amount of data has been reported, the preparation process of nanotechnology is still complex, and there is little data on the stability of these preparation processes.

Based on the extensive demand for functional food, nano functional food is a very active research field. Nanoparticle delivery system can effectively improve the activity and absorption and utilization of active ingredients in vivo, which can provide a promising strategy for the development of functional food. However, there is still a long way to go for the transformation of nano functional food from the laboratory to industrialization. In future research, the safety of carrier materials, the number of auxiliary materials, and the feasibility of preparation process and storage stability should be continuously studied. If nano functional food can overcome these challenges, more and more effective nano functional foods will be developed reasonably.

## Figures and Tables

**Table 1:** Examples of nanoliposomes for functional food nanoparticle delivery systems.

Materials	Method	Active Components	Size (nm)	Zeta-Potential (mV)	Application	References
Soybean oil	Supercritical fluid technology	Peppermint essential oil	35.7	-	Atibacterial agent	[27]
Gelucire 50/30	Spray drying	Lippia sidoides essential oil	525.3	−25.9	Encapsulates essential oils	[28]
Palmitic acid, lecithin	Micro-emulsion method	Anthocyanin	455	-	Antioxidant	[29]
Lecithin	Ultrasonic emulsification	Astaxanthin	94	-	Food additives	[30]
Lecithin	Hot homogeneous method	Vitamin B12	200	−5.28	Anti-cancer drug	[31]
Soybean lecithin	Sonication method	DHA	130	-	Food stabilizers	[32]
